# Chemical profile and *in vivo* hypoglycemic effects of *Syzygium jambos*, *Costus speciosus* and *Tapeinochilos ananassae* plant extracts used as diabetes adjuvants in Puerto Rico

**DOI:** 10.1186/s12906-015-0772-7

**Published:** 2015-07-22

**Authors:** Jannette Gavillán-Suárez, Alexandra Aguilar-Perez, Natalie Rivera-Ortiz, Karla Rodríguez-Tirado, Wanda Figueroa-Cuilan, Lorelein Morales-Santiago, Gerónimo Maldonado-Martínez, Luis A. Cubano, Michelle M. Martínez-Montemayor

**Affiliations:** Institute of Interdisciplinary Research, University of Puerto Rico at Cayey, PO Box 372230, Cayey, 00737-2230 Puerto Rico; Department of Chemistry, University of Puerto Rico at Cayey, PO Box 372230, Cayey, 00737-2230 Puerto Rico; Department of Biochemistry, Universidad Central del Caribe-School of Medicine, P.O. Box 60327, Bayamón, 00960-6032 Puerto Rico; Present address: University of Puerto Rico Medical Sciences Campus, PO Box 365067, San Juan, 00936-5067 Puerto Rico; Present address: University of Missouri-Columbia, Division of Biological Sciences, 110 Tucker Hall, Columbia, MO 65211-7400 USA; Data Management & Statistical Research Support Unit, Universidad Central del Caribe-School of Medicine, P.O. Box 60327, Bayamón, 00960-6032 Puerto Rico; Department of Anatomy and Cell Biology, Universidad Central del Caribe-School of Medicine, P.O. Box 60327, Bayamón, 00960-6032 Puerto Rico

**Keywords:** Diabetes adjuvants, C57BLKS/J *db/db* and C57BL/J *ob/ob* mice, Chemical profile, *Syzygium jambos*, *Tapeinochilus ananassae*, *Costus speciosus*

## Abstract

**Background:**

The increasing numbers of people who use plant-based remedies as alternative or complementary medicine call for the validation of less known herbal formulations used to treat their ailments. Since Puerto Rico has the highest rate of Type 2 diabetes within all the states and territories of the United States, and Puerto Ricans commonly use plants as diabetes adjuvants, it is important to study the plants’ physiological effects, and identify their bioactive compounds to understand their role in modulation of blood glucose levels. We present the phytochemical profiles and hypoglycemic effects of *Tapeinochilus ananassae*, *Costus speciosus* and *Syzygium jambos*.

**Methods:**

Phytochemicals in methanolic and aqueous extracts were analyzed by thin layer chromatography (TLC). Alkaloids (Bromocresol green, λ = 470 nm), flavonoids (AlCl_3_, λ = 415 nm), saponins (DNS, λ = 760 nm), tannins (FeCl_3_/K_4_Fe(CN)_6_, λ = 395 nm) and phenolics (Folin-Ciocalteau, λ = 765 nm) were quantified. Male C57BLKS/J (*db/db*) and C57BL/J (*ob/ob*) genetically obese mice were orally gavaged with aqueous extracts of lyophilized plant decoctions for 10wks.

**Results:**

Our results show that *T. ananassae* had significantly greater amounts of flavonoids and tannins, while *S. jambos* showed the greatest concentration of phenolics and *C. speciosus* exhibited higher amounts of alkaloids. C57BLKS/J *db/db* treated with plant extracts show better glucose modulation when the extracts are administered in complement with an insulin injection. Finally, C57BL/J *ob/ob* mice on *T. ananassae* and *S. jambos* treatments show better blood glucose modulation over time.

**Conclusion:**

These results document for the first time the chemical profile of *T. ananassae* and provide evidence for a potential anti-diabetic efficacy of *T. ananassae* and *S. jambos*.

**Electronic supplementary material:**

The online version of this article (doi:10.1186/s12906-015-0772-7) contains supplementary material, which is available to authorized users.

## Background

The widespread use of medicinal plants to mitigate or cure medical conditions such as diabetes calls for their scientific investigation. According to the Annual Survey by the Centers for Disease Control and Prevention (CDC) in Atlanta; since 1996, Puerto Rico has the highest Type 2 diabetes rate within the United States in individuals 18 years or older [1, 2]. After conducting an ethnopharmacological survey in 11 municipalities of southeast Puerto Rico and carrying out a literature review about herbal remedies used to manage hyperglycemia, we identified that people living in the southeast rural region of Puerto Rico frequently use four medicinal plants as alternative or complementary treatments for diabetes: *Costus speciosus* (J. Koening) Sm. (Zingiberaceae), *Syzygium jambos* (L.) Alston (Myrtaceae), *Tradescantia spathacea* and *Tapeinochilos ananassae* K. Schum. (Zingiberaceae) [3–5]. *In vitro* studies using these plants to screen their activities as inhibitors of processes related to the physiopathology (oxidative stress, protein glycosylation and aldose reductase activity) of diabetes (data not shown) led us to perform chemical profiling and functional *in vivo* studies of *C. speciosus, S. jambos* and *T. ananassae*.

*C. speciosus*, a small plant with scarlet flowers, commonly known as “insulin” in Puerto Rico, is a plant widely used in indigenous systems of medicine for the treatment of various ailments. *C. speciosus* rhizomes are used as an alternative source for diosgenin, and also to control diabetes due to the antidiabetic, antilipidemic and antioxidant effects of its compounds including sesquiterpene lactones [6–8]. *S. jambos*, “rose apple”, is an ornamental fruit tree from Southeast Asia that is also found in tropical regions. This medicinal plant is used to treat diabetes, inflammation, and gastrointestinal disorders [9, 10]. Diabetic animals treated with aqueous and ethanolic extracts from the seeds of *S. jambos* show reduced glycemic effect [11]. *S. jambos* leaf and fruit extracts contain high concentrations of tannins and phenolic acids, respectively [12, 13]. Another plant commonly referred to as “insulin” by populations of southeast Puerto Rico is *T. ananassae*, an ornamental garden plant native to Malasia, Indonesia, New Guinea and Australia. There are no previous studies describing *T. ananassae*’s phytochemical profile or anti-diabetic activity, although we previously reported that this plant is currently being used as a herbal remedy for diabetes in the southeast region of Puerto Rico [5].

The beneficial effects of diverse groups of phytochemicals in herbal remedies have been related to activities consistent with their potential use in treating diabetic disorders and complications. The *in vivo* anti-diabetic activity of plant extracts has been correlated with their flavonoid and total phenolic content [14, 15]. Glycosides, flavonoids, tannins and alkaloids have shown reliable activities that may be useful for the treatment of Type 2 diabetes [16]. Also saponins, such as oleanolic acid, exhibit hypoglycemic activity and resveratrol, a phenolic compound, shows insulin-like effects in streptozotocin-induced diabetic rats [17, 18]. In the case of tannins, two modes of action have been proposed to explain their anti-diabetic potential. At the protein level, tannins act via insulin receptor activation leading to an increase in glucose uptake rate and lower glucose levels. At the molecular level, tannins have significant superoxide scavenging and antioxidant activity [19]. These facts are relevant since high levels of superoxide ions in pancreatic β-cells, block insulin signaling, affecting glucose regulation [20].

Although the traditional use of decoctions of *C. speciosus* and *S. jambos* has been reported as both complementary and alternative treatments for diabetes, the antidiabetic effects of the decoctions of these plants, including *T. ananassae* have not been validated in animal models. Consequently, this report presents the results of *in vivo* studies using C57BLKS/J *db/db* and C57BLKS/J *ob/ob* genetic diabetes animal models. Moreover, we present the systematic characterization of major phytochemicals and potential markers of anti-diabetic activity in the plant aqueous extracts.

## Methods

### Chemicals and reagents

Folin-Ciocalteu reagent, HPLC grade methanol, tannic acid ACS reagent, Quillaja saponin, 3,5-dinitrosalicilic acid (98 %), aluminum trichloride, sulfuric acid (18 M), glucose (99.5 %), stigmasterol (95 %), hydroquinone (99 %), ursolic acid (98.5 %), digitoxin (92 %) and bromocresol green (95 %) were obtained from Sigma-Aldrich (St. Louis, MO, USA). Quercetin dihydrate (98 %) and dragendorff’s reagent were obtained from Aldrich (Milwaukee, WI, USA). Ferric Chloride (FeCl_3_•6H_2_O) (97.0-102.0 %) was obtained from Spectrum Chemical and Lab Products (Gardena, California, USA) and potassium ferrocyanide (K_4_Fe(CN)_6_•3H_2_O), HCl (12 M), sodium hydrogen phosphate, ethyl acetate (EtOac), acetic anhydride and dichloromethane were obtained from Thermo Fisher Scientific (New Jersey, USA). Nicotine (99 %) was obtained from VWR (New Jersey, USA). All chemicals were used without further purification.

### Ethnopharmacological survey

The questionnaire used in the ethnopharmacological survey in Puerto Rico was adapted from the one published by TRAMIL Network (www.tramil.net). The second section of the questionnaire follows a structured interview, asking participants to provide information about the botanical remedies used by the family as the first treatment for the ailments included in the survey. Open-ended questions were used to obtain a detailed description of the health problem, treatment preparation, application and results obtained, including dosage and contraindications or side effects for adults and children. Plants having a usage frequency of 20 % or more for a particular ailment were selected for the *in vitro* or *in vivo* studies. The Institutional Review Board at the University of Puerto Rico-Cayey approved the questionnaire used for the survey and the informed consent forms. After completing the survey and reviewing the ethnobotanical literature for herbal remedies used for their hypoglycemic effects, medicinal plants used as diabetes adjuvants were identified [3–5].

### Plant collection and identification

Leaves of *C. speciosus*, *S. jambos* and *T. ananassae* were collected in Puerto Rico. Vouchers of *Costus specious* (019660), *Tapeinochilus ananassae* (019553), *Syzygium jambos* (019663) were numbered and deposited at the George Proctor Herbarium (SJ) in Puerto Rico. José Sustache, Botanist and Head of the PR Department of Natural and Environmental Resources classified the botanical species.

### Preparation of decoctions, methanolic and aqueous extracts

For *in vitro* and quantitative analysis, fresh leaves (50 g) were weighed, freeze-dried using a Freezone 4.5 lyophilizer and extracted overnight with methanol (MeOH) (350 mL) using a Soxhlet apparatus. The resulting extracts were concentrated to dryness by rotatory evaporation (Yamato RE-200) at room temperature to yield from 1 – 11 % w/w of extract (1-5 g extract/ 45 – 200 g fresh leaves). Stock solutions of dry plant extracts were re-dissolved in various solvents (depending on the phytochemical to be tested). Alternatively, for the preparation of decoctions used in *in vitro, in vivo* and quantitative analysis, fresh leaves (30 g) were boiled in 100 mL of distilled, deionized water, concentrated to 15 mL, to lyophilize 3 × 5 mL replicates, filtered through cheesecloth and freeze-dried. The resulting solids were re-dissolved in ddH_2_O and used to prepare aqueous extracts of known concentrations. All extracts were filtered using a 0.4 μm Nanopure® filter syringe before assayed [21].

### Dosage calculation

A tea of *C. speciosus* leaves was prepared according to a consensus of dosage and administration reported during the TRAMIL interviews from Puerto Ricans who self-medicate with this medicinal plant [5]. The preparation consisted of boiling 18.00 g of fresh leaves (six medium size leaves) in 1.89 L of ddH_2_O for 15 min. The resulting extract was filtered through cheesecloth and aliquots of 5-10 mL were freeze-dried to calculate the total solids. The %w/w yield was 6.6 %, which corresponds to 1.14 g of total solids in 1.89 L. According to the survey the daily dosage of the tea is 250 mL (8 oz) or 158.3 mg of tea solids. Assuming an average body weight of 70Kg, the dose of tea solids per Kg of body weight is 2.3 mg/kg_BW, which was the dose used as reference for preparing the remaining teas and used in all *in vivo* studies.

### Chromatographic separation and qualitative analysis

Thin layer chromatography was performed using 10 × 10 cm, 0.2 mm Nano-silica gel 60 HPTLC plates (Supelco, PA). Lyophilized methanolic and aqueous extracts were dissolved in methanol or methanol:water, respectively according to procedures described previously [22–24]. The following solvent systems and visualization techniques were used to obtain the phytochemical profile as alkaloids (dichloromethane (CH_2_Cl_2_):MeOH (15:1); 254 nm and visible with Dragendorff’s reagent), cardiac glycosides (EtOAc:MeOH:water (20:2.5:2.5); 366 nm after spraying with sulphuric acid), phenolics (CH_2_Cl_2_:EtOAc: glacial acetic acid (5:4:1); visible with Folin-Ciocalteu’s reagent and heating), saponins (CH_2_Cl_2_:glacial acetic acid:MeOH:water (16:8.5:3:2); 366 nm and visible light with anisaldehyde-sulphuric acid reagent), sterols (CH_2_Cl_2_:glacial acetic acid:MeOH:water (32:17:6:4); visible light with FeCl_3_:acetic acid:sulfuric acid solution and heating), tannins, hydrolysable tannins, flavonoids and terpenoids (EtOAc:toluene:acetic acid (8:4:1); 254, 366 nm and visible with anisaldehyde-sulphuric acid reagent and heating). Appropiate standards were used for each class of phytochemicals (Table 1). The fluorescence of each spot was noted and Rf values were determined.

**Table 1:** Phytochemical analysis by TLC of plant extracts^a^

Plant extract	Phytochemicals^b^ (Visible and UV fluorescence)							
	Flavonoids (orange, yellow)	Alkaloids (pink-orange)	Phenolics (dark blue)	Saponins (orange)	Sterols (purple)	Terpenoids (violet-pink)	Cardiac glycosides (orange)	Tannins (beige)
*T. ananassae* (met)	0.34, 0.41, 0.48	0.21, 0.30, 0.69, 0.84	0.21, 0.54, 0.81	0.93	0.78, 0.91	0.18	0.92	ND
*S. jambos* (met)	0.33, 0.37	0.15, 0.25, 0.31, 0.38	0.11, 0.33, 0.38, 0.43, 0.54, 0.56, 0.64, 0.70, 0.84, 0.90	0.36, 0.92	0.86, 0.95	0.05	0.91	0.79
*C. speciosus* (met)	ND	0.04, 0.32, 0.50, 0.56, 0.63, 0.73, 0.81	0.62, 0.78, 0.89	0.96	0.85, 0.94	0.14	0.91	0.78
*T. ananassae* (aq)	-	ND	0.36	0.67	-	ND	-	0.10-0.17^d^
*S. jambos* (aq)	-	ND	0.06, 0.70-0.80	0.36, 0.52, 0.89	0.94	-	-	ND
*C. speciosus* (aq)	0.44-0.55^c^ 0.51-0.59	ND	0.06, 0.09, 0.20	0.66	0.95	ND	-	ND

^a^Rf values reported as the means of two chromatograms; ^b^Standards: Quercetine (0.12, yellow-orange), Caffeine (0.55, light pink) and Nicotine (0.34, light pink), Hydroquinone (0.09, dark blue), Commercial saponin (0.28, orange), Stigmasterol (0.97, purple) and Ursolic acid (0.15, violet-pink), Digitoxin (0.75, orange), Tannic Acid (0.06, beige); ^c^Yellow at 366 nm; ^d^Yellow at 254 nm; *ND* Not detected

### Quantitative phytochemical analysis

Total phenolic content (TPC) in methanolic and aqueous extracts dissolved in DMSO was determined using Folin-Ciocalteu method (765 nm) with some modifications, while flavonoids in methanolic extracts were determined by the aluminum chloride method (415 nm) using quercetin as standard, as described by [25]. The bromocresol green method [26] and the Prussian Blue reaction [27] were used to quantify total alkaloids (470 nm) and tannins (395 nm) in methanolic and aqueous extracts using nicotine and tannic acid as standards, respectively. Saponins were determined by the dinitrosalicylic acid (DNS) method (540 nm) as reported previously [17, 28].

### Animals

#### Study 1

Male BKS.Cg-Dock7m +/+ Leprdb/J (C57BLKS/J *db/db*) 5 wks old mice (Jackson Laboratories, ME) were maintained under a constant 12-h light:12-h dark cycle with a temperature of 22–24 °C. Four to five animals per cage were divided into 12 treatment groups, for a total of 50 mice. The mice had free access to NIH#31 M Rodent Diet pellets (Agricultural Exports Inc., Doylestown, PA) and water. Consumption of food and water were monitored daily. Mice were weighed weekly for 10 wks.

#### Study 2

Male B6.Cg-Lepob/J (C57BL/J *ob/ob*), 5 wk old mice (Jackson Laboratories, ME) were maintained as those described in Study 1. Three to four animals per cage were divided into 4 treatment groups (n = 11 or 12 per treatment). The Institutional Animal Care and Use Committees at Universidad Central del Caribe-School of Medicine approved the protocols for Studies 1 and 2.

### Treatment administration

#### Study 1

*db/db* mice were orally gavaged daily for 10 wks with control (sterile water) or 0.2, 2.2, 22 mg/kg_BW) in a 100 μL volume of *T. ananassae*, *C. speciosus* and *S. jambos* aqueous extracts or 220 mg/kg_BW in a 100 μL volume of *T. ananassae*, and *C. speciosus* aqueous extracts, for a total of 12 treatments.

#### Study 2

*ob/ob* mice were orally gavaged daily for 10 wks with control (sterile water) or 2.2 mg/kg_BW in a 100 μL volume of *T. ananassae*, *C. speciosus* and *S. jambos* aqueous extracts.

### Glucose tolerance test (IP-GTT)

**Fig. 1:**
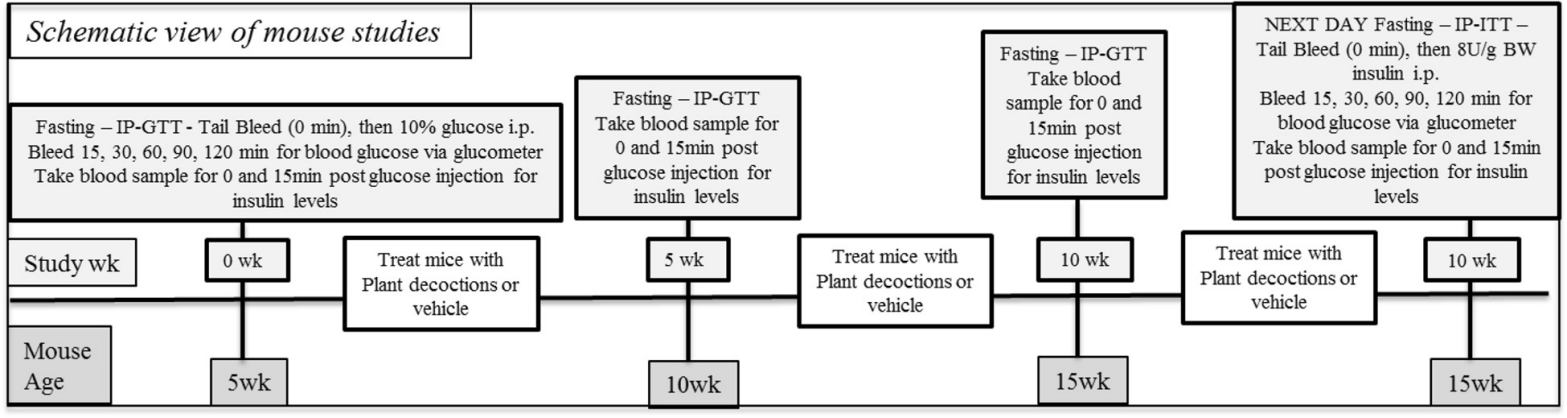
Schematic drawing of *in vivo* experimental procedures. In study 1 and study 2, the mice (5 wks old) were randomly assigned to the various experimental treatments that were administered daily for 10 wks. An IP-GTT was performed at 0, 5 and 10 wks of the study, while an IP-ITT was performed at 10 wks. In each intervention blood was collected at 0 and 15 min post injection to detect insulin plasma levels

IP-GTT was performed after a 12-h fast as depicted in Fig. 1. A 10 % glucose solution was injected i.p. (1 mg/g BW) and blood samples were obtained from the tail vein at 0, 15, 30, 60, 90, and 120 min after glucose administration as in [29]. Mice were tail bled at 0 wks (before treatment intervention), at 5 wks and at 10 wks (end of the study). Blood glucose was determined using a glucometer (Optium EZ, Abbot Laboratories and One Touch Ultra Mini, Johnson and Johnson) via test strips. Limit of detection of Optium EZ is 500 mg/dL (used in Study 1) and of One Touch Ultra Mini is 600 mg/dL (used in Study 1 and 2).

### Insulin tolerance test (IP-ITT)

IP-ITT was performed at the end of the study to determine the effects of insulin on blood glucose modulation between control and treatment groups as depicted in Fig. 1. The *db/db* mice become severely diabetic by 4 wks of age as described by Buchanan [30] thus we used an insulin (Humulin R U-500, Lilly, Indianapolis, IN) dose of 8units/g BW to achieve adequate glucose lowering. Mice were fasted (12-h), on a different day than that of the IP-GTT; they were tail bled pre-injection and then glucose concentrations were determined at 15, 30, 60, 90, and 120 min post-injection as by Keller et al. [29].

### Plasma insulin detection

Plasma insulin levels were analyzed using the Ultrasensitive Mouse Insulin ELISA Kit (Crystal Chem Inc., IL) following manufacturer’s instructions for the wide range analysis. Five microliters of sample were used; the assay was conducted in duplicates. For Study 1 we used plasma samples collected at 10 wks of control and of *S. jambos* treated mice at a concentration of 2.2 mg/kg_BW at the IP-GTT and IP-ITT at 0 or 15 min after glucose or insulin injection, respectively (see Fig. 1). For study 2 we used plasma samples collected at 10 wks of *S. jambos* and *T. ananassae* treated mice compared to controls at the IP-GTT and IP-ITT at 0 or 15 min after glucose or insulin injection, respectively (see Fig. 1). The plates were read using the Glomax Multi Detection System (Promega, WI) at an absorbance of 450 nm.

### Statistical analysis

All *in vitro* tests were performed in triplicate. Results are expressed as means ± standard error of the mean. The concentrations of phytochemicals obtained from the quantitative analyses were estimated from the least-squares regression lines (r^2^ 98.99 – 1.00 %). *In vitro* statistical analyses were done using GraphPad Prism version 5.0b (San Diego, CA). The data were analyzed using regular one-way analysis of variance procedures. Values *P* < 0.05 were considered significant. For *in vivo* studies, to assess the behavior of all the variables in our models, a normality diagnostic was performed. A multivariate analysis was conducted, in particular; a repeated measures ANOVA model was constructed in order to establish the statistical association between the dependent variables and each study variable. A Mauchly’s test of sphericity was performed to assess if our models had or not the assumption of compound symmetry. If non-significant, we report the univariate results with an Epsilon correction; if significant, we report the multivariate results using the Pillai’s Trace estimator. Either of the last explained results was used to evaluate the time effect in our models. A test of Between-Subjects Effect with a Pairwise Comparisons (Bonferroni’s or Dunnett’s adjustment for multiple comparisons) was applied to perceive statistical differences between and within the groups. The analyses were constructed using a general to specific approach as follows: analysis WHOLE, where we confronted all weeks (0, 5 & 10 altogether), analysis at 0, 5 and at 10 wks. The sub-measures were 0, 15, 30, 60, 90, 120 min. Finally; we constructed various plots to help illustrate the changes over time of our studied groups. Due to the exponential nature and intrinsic unstable behavior of the dependent variable (blood glucose levels) over time, a percent change transformation was made in the form of %∆ = New Value – Old Value /(ABS) Old Value × 100. Therefore, if the original value increases, the percent change decreases and vice versa. The significance level (α) was set to ≤ 0.05. The statistical software for *in vivo* analysis used was Statistical Package for Social Sciences (SPSS, Chicago, IL) v.17.0 for Windows.

## Results

### Profile of surveyed population using medicinal plants

Table 2 shows the profile of the surveyed population that uses herbal remedies as the first treatment for the ailments assessed during the ethnopharmacological survey. A total of 10 out of 118 (8.5 %) families in the study population reported using herbal remedies for diabetes as alternative or complementary treatments. The median age of the women who reported the use of medicinal plants for diabetes was 51.1 years (±16.7 years). The majority of the participants reported to have college education and being married (70 % in both cases). Roughly, half of the study population that uses medicinal plants for diabetes reported being employed. Nine different plant remedies were used among the study population the last time that one of the family members was treated for diabetes. The more frequent descriptions of diabetes as a health problem were uncontrolled sugar levels (high or low) and dizziness. Other descriptions included dry mouth, excessive thirst, sleepiness, restlessness, not feeling the legs, blindness or poor vision and frequent urination. About half of the treatments were reported once (55 %) and the use was classified as not significant according to TRAMIL methodology [31]. Most of the plants (60 %) were obtained at the store or market. Decoction of fresh leaves was the most frequent mode of preparation of the herbal remedies. Dosages were variable, with most families reporting the use of one cup of tea (decoction) daily or during one week. The herbal remedies prepared from *Costus speciosus* (J. Koening) Sm. (Zingiberaceae) and *Tapeinochilos ananassae* K. Schum. (Zingiberaceae) were selected for the present study as their use was significant according to TRAMIL and they are commonly known as “insulin” in the studied population.

**Table 2:** Profile of surveyed population using medicinal plants

Indicator/Variable	Results
Use of herbal remedies for diabetes	10/118 families (8.5 %)
Median age of women	51.1 years ± 16.7 years
Education and marital status	70 % college education and married
Employment status	50 % employed
Frequency of reported treatments	55 % once/surveyed population

### Major groups of phytochemicals were found in plant extracts

To establish product integrity during the scientific investigation of complex botanical products, the National Institutes of Health (NIH), National Center for Complementary and Integrative Health (NCCIH) has published guidelines that required the characterization (chemical profile or fingerprint) and identification of relevant marker compounds used for standardization [32]. Qualitative TLC analysis of methanolic and aqueous extracts of the leaves of *S. jambos*, *T. ananassae* and *C. speciosus* showed the presence of flavonoids, alkaloids, phenolic compounds, saponins, terpenoids, tannins and cardiac glycosides (Table 1). Aqueous extracts showed fewer phytochemicals than methanolic extracts. TLC analysis confirmed the presence of a significant number of phenolics in *S. jambos* methanolic and aqueous extracts, alkaloids in *C. speciosus* methanolic extracts, flavonoids in *T. ananassae* methanolic extracts and *C. speciosus* aqueous extracts, and tannins in *T. ananassae* aqueous extracts. The results for the quantitative analysis of the extracts presented in Table 3, confirms the qualitative chemical profile and shows a significant concentration of phenolics in *S. jambos* (0.45 mg QE/mg extract), flavonoids and tannins in *T. ananassae* (24.3 mg QE/g DW and 3.361 mg Tannic acid/g FW, respectively) and alkaloids in *C. speciosus* dry or fresh leaves (806.0 % μg Nicotine/mg FW).

**Table 3:** Quantitative analysis of plant extracts

Plant Extract	Flavonoids (±SD mg QE/g DW)	Total Phenolic Content (± SD mg QE/mg extract)		Tannin Content (± mg Tannic acid eq/g FW)	Saponins (± SD % mg Quillaja/mg DW	Alkaloids (± SD % μg Nicotine eq/mg FW)	
		Methanolic	Aqueous			Methanolic	Aqueous
*S.jambos*	24.3 ± 1.3^a^	0.45 ± 0.09^a^	0.18 ± 0.04^a^	3.361 ± 0.004^a^	0.6 ± 0.3	nd^a^	1.80 ± 0.10^a^
*T. ananasse*	29.8 ± 0.0^b^	0.14 ± 0.02^b^	0.034 ± 0.005^b^	10.337 ± 0.008^b^	0.4 ± 0.4	6.90 ± 0.17^b^	5.20 ± 0.43^b^
*C. speciosus*	15.8 ± 0.9^c^	0.35 ± 0.01^a^	0.060 ± 0.002^c^	1.516 ± 0.008^c^	0.2 ± 0.3	806.0 ± 172.0^c^	3.49 ± 0.15^c^

*ND* Not detected. Values are mean ± SD. Different letters show significant differences, *P* < 0.05

### Plant extracts do not affect weight gain, food or water intake in C57BLKS/J (*db/db*) and C57BL/J (*ob/ob*) mice

In Study 1, three out of the 50 mice died from causes unrelated to treatment toxicity or to an unsuccessful gavage. Results show that there is no significant effect of treatment on weight gain, average food or water intake throughout the 10 wks of the study, an indication that the extracts were not toxic to mice (Additional file 1: Figure S1). In Study 2, none of the *ob/ob* mice died and there was no significant effect of treatments on weight gain, or on average food or water intake. (Additional file 2: Figure S2). Interestingly, overall *db/db* mice weighed less, and consumed more water than *ob/ob* mice.

### Plant decoctions modulate C57BLKS/J (*db/db*) mice blood glucose levels when administered in complement to insulin

**Fig. 2:**
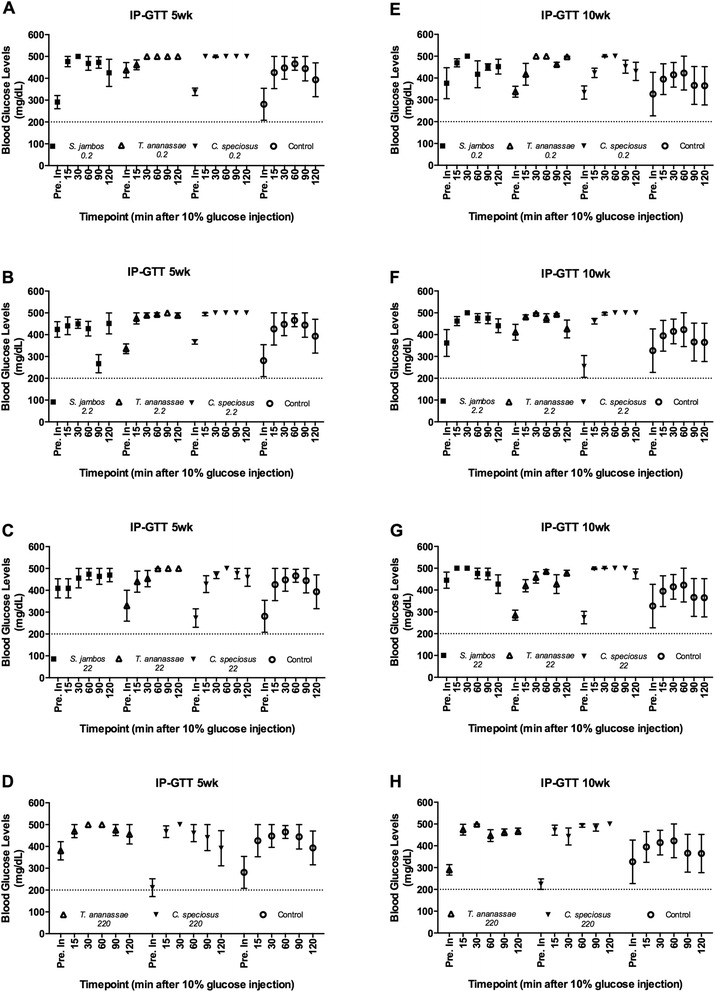
IP-Glucose Tolerance Test (IP-GTT) in the C57BLKS/J (*db/db*) mouse model. A-D. IP-GTT performed at 5 wks post-treatment with (**a**) 0.2 mg/kg_BW, (**b**) 2.2 mg/kg_BW, (**c**) 22 mg/kg_BW of *S. jambos, T. ananassae, C. speciosus* aqueous extracts or water (control), (**d**) 220 mg/kg_BW of *T. ananassae, C. speciosus* aqueous extracts or water (control). E-H. IP-GTT performed at 10 wks - post-treatment with (**e**) 0.2 mg/kg_BW, (**f**) 2.2 mg/kg_BW, (**g**) 22 mg/kg_BW of *S. jambos, T. ananassae, C. speciosus* aqueous extracts or water (control), (**h**) 220 mg/kg_BW of *T. ananassae, C. speciosus* aqueous extracts or water (control). Horizontal dashed line is set at 200 mg/dL. Mean values of BGL before (Pre inj.) and after 15, 30, 60, 90 and 120 min of an IP 10 % glucose solution injection. Values are means ± SEM, n = 4-5/group

**Fig. 3:**
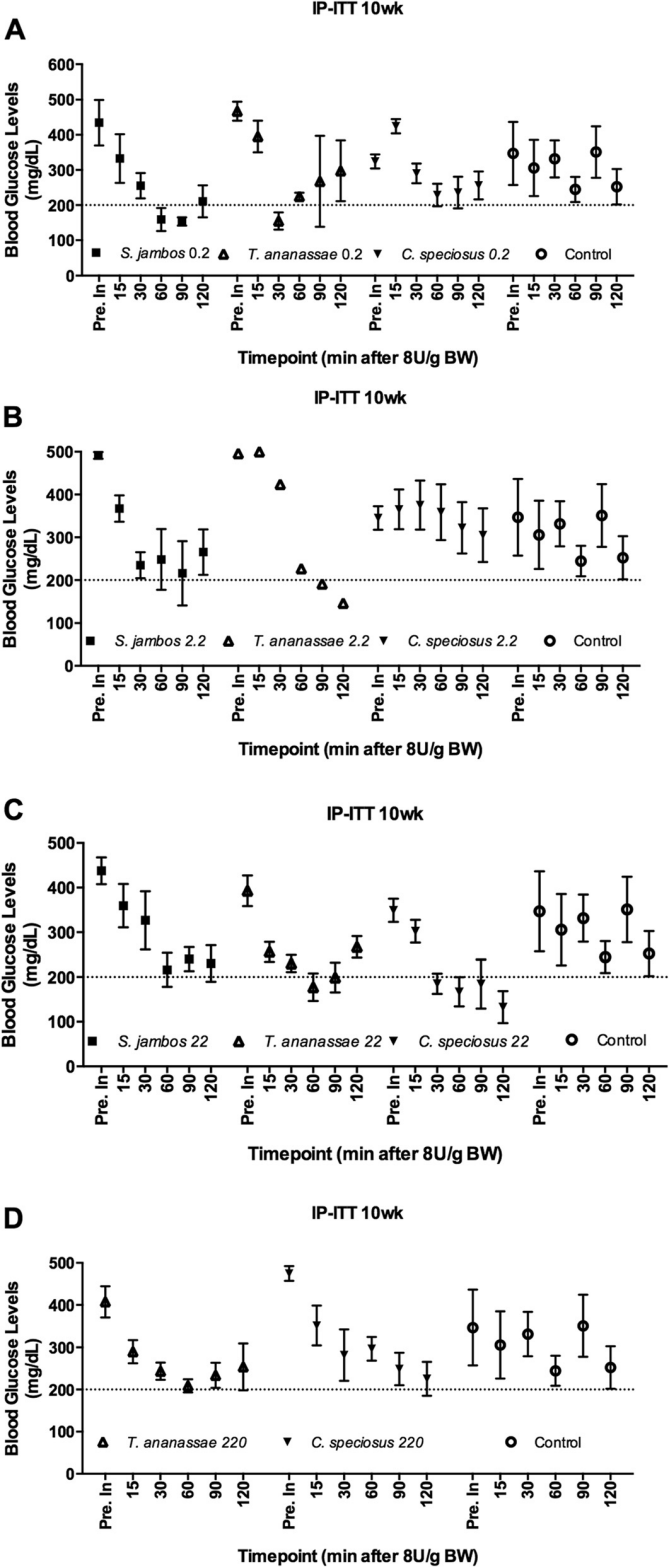
IP-Insulin Tolerance Test (IP-ITT) in the C57BLKS/J (*db/db*) mouse model. **a**-**d** IP-ITT performed at 10 wks - post-treatment with 0.2 mg/kg_BW (**a**), 2.2 mg/kg_BW (**b**), 22 mg/kg_BW of *S. jambos, T. ananassae, C. speciosus* aqueous extracts or water (control) (**c**), 220 mg/kg_BW (**d**) of *T. ananassae, C. speciosus* aqueous extracts or water (control). Horizontal dashed line is set at 200 mg/dL. Mean values of BGL before (Pre inj.) and after 15, 30, 60, 90 and 120 min of an IP insulin (8U/g BW) injection. Values are means ± SEM, n = 4-5/group

Blood glucose levels (BGL) were monitored in mice treated with plant extracts or control (total of 12 treatments) at 5 and 10 wks post-treatment. In Study 1, there was no significant effect of treatments in lowering blood glucose levels at 5 or 10 wks post-treatment. At 5 wks, *S. jambos* treated mice showed better blood glucose modulation compared to the mice receiving other plant extract treatments (Fig. 2a-d). Mice on *C. speciosus* 22 (BGL 273 mg/dL), and 220 mg/kg_BW (BGL 211 mg/dL) had lower baseline BGL compared to vehicle treated mice. After the glucose injection, most of the animals treated with *C. speciosus* 2.2 mg/kg_BW had glucose levels greater than 500 mg/dL and were not able to reduce their BGL to basal state, indicating that glucose tolerance in these mice is impaired (Fig. 2b). Moreover, mice that received *C. speciosus* 220 mg/kg_BW, tended to show lower BGL (BGL 392 mg/dL) when compared to the animals on the control treatment at the last reading (120 min, BGL 467 mg/dL). Interestingly, mice treated with *S. jambos* 2.2 mg/kg showed decreased BGL at 90 min (BGL 267 mg/dL) post injection. These results suggest that mice receiving 2.2 mg/dL of *S. jambos* display gradual blood glucose modulation (increased and decreased) peaks*.* At 10 wks, baseline BGL of mice on *C. speciosus* 2.2, 22, 220 mg/kg_BW, and *T. ananassae* 2.2 mg/kg_BW were lower compared to control mice (Fig. 2e-h). Furthermore, at 60 min post-glucose mice on all *S. jambos* treatments had lower blood glucose levels compared to *C. speciosus* mice, and by 120 min, all of the glucose readings for *T. ananassae* and *S. jambos* mice were below the limit of detection of the glucometer (500 mg/dL). Interestingly, after 15 min of glucose administration plasma insulin levels were significantly higher (*P <* 0.05) in both control and *S. jambos* 2.2 mg/kg_BW treated mice at 10 wks post-treatment (Fig. 5a). The IP-ITT data showed that baseline BGL for all mice were in a range from BGL 325 to 491 mg/dL (Fig. 3a-d). By 60 min most of the treated mice showed a drop in BGL where the lowest blood glucose value obtained was BGL 159 mg/dL (*S. jambos* 0.2 mg/kg_BW), followed by BGL 167 mg/dL (*C. speciosus* 22 mg/kg_BW), and BGL 177 mg/dL (*T. ananassae* 22 mg/kg_BW). The remaining plant treated and control (BGL 245 mg/dL) mice had values greater than 200 mg/dL at 60 min, while at 120 min the lowest BGL were 122 mg/dL (*C. speciosus* 22 mg/kg_BW), followed by BGL 146 mg/dL (*T. ananassae* 2.2 mg/kg_BW). The remaining mice displayed BGL over 200 mg/dL; however mice treated with *C. speciosus* 220 mg/kg_BW, and *S. jambos* 0.2 and 22 mg/kg_BW displayed lower values than those of the control mice (BGL 252 mg/dL) at 120 min post-injection. Plasma insulin levels for the control and *S. jambos* 2.2 mg/kg_BW were similarly affected during the IP-ITT after the insulin injection (Fig. 5b).

### *T. ananassae* (5 wks) and *S. jambos* (5 and 10 wks) significantly reduce BGL in *ob/ob* mice

**Fig. 4:**
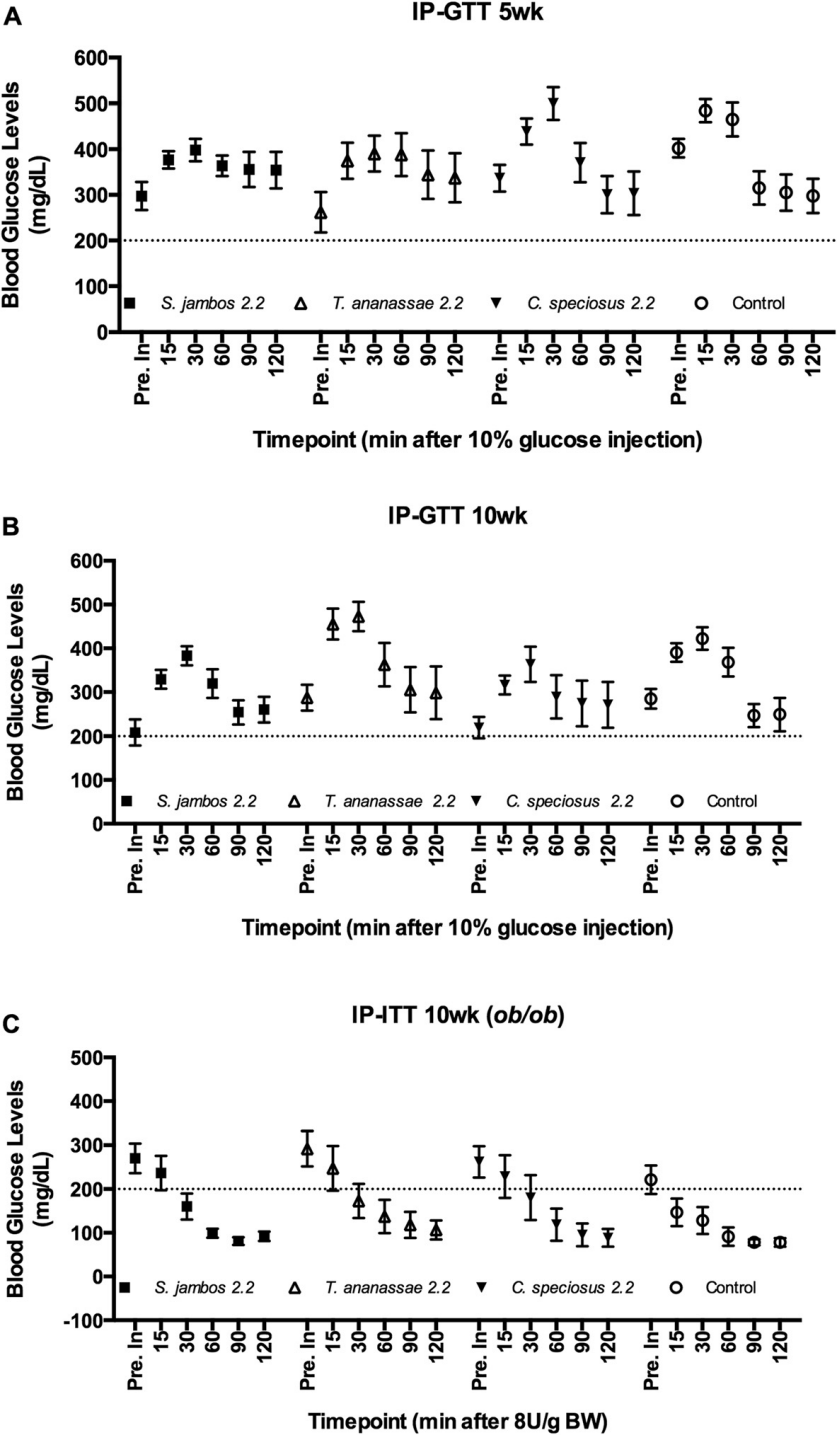
IP-GTT and IP-ITT in the C57BL/J (*ob/ob*) mouse model. A-C. IP-GTT performed at 5 wks (**a**) and 10 wks (**b**, **c**) post-treatment with 2.2 mg/kg of *S. jambos, T. ananassae, C. speciosus* aqueous extracts or water (control). Horizontal dashed line is set at 200 mg/dL. Mean values of blood glucose levels before (Pre inj.) and after 15, 30, 60, 90 and 120 min of an IP 10 % glucose solution (**a**, **b**) or insulin (8U/g BW) (**c**) injection. Values are means ± SEM, n (Control, *C. speciosus, T. ananassae*) = 11/group, n (*S. jambos*) =12/group

**Fig. 5:**
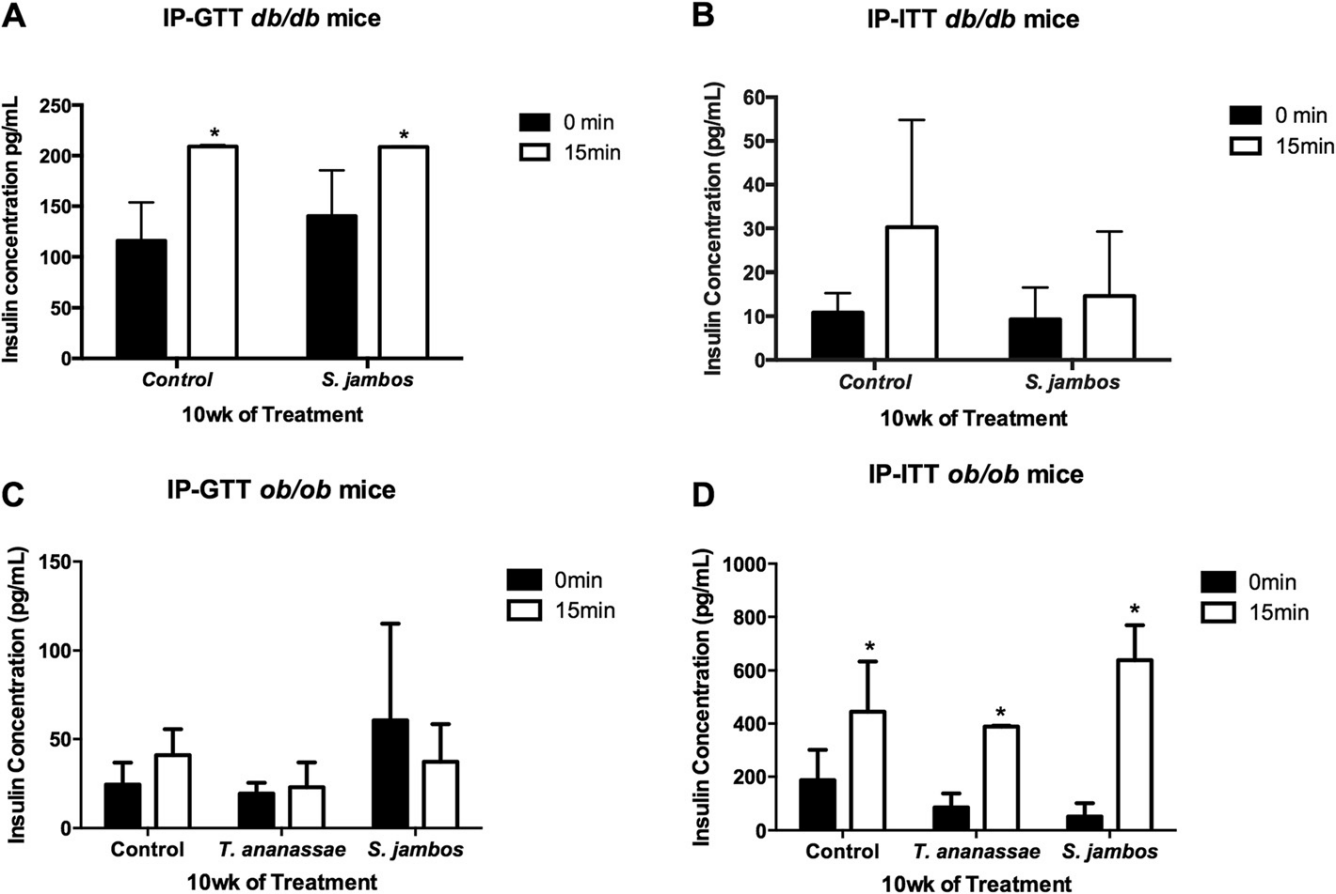
Plasma insulin levels of mice treated with plant decoctions. **a** Plasma insulin levels of *db/db* mice before (0 min) and after (15 min) a 10 % glucose injection 10 wks post-treatment with water (Control) or 2.2 mg/kg_BW *S. jambos*, aqueous extracts. **b** Plasma insulin levels of *db/db* mice before (0 min) and after (15 min) a 8U/g BW insulin injection 10 wks post-treatment with water (Control) or 2.2 mg/kg_BW *S. jambos*, aqueous extracts. **c** Plasma insulin levels of *ob/ob* mice before (0 min) and after (15 min) a 10 % glucose injection 10 wks post-treatment with water (Control) or 2.2 mg/kg_BW *S. jambos or T. ananassae*, aqueous extracts. **d** Plasma insulin levels of *ob/ob* mice before (0 min) and after (15 min) a 8U/g BW insulin injection 10 wks post-treatment with water (Control) or 2.2 mg/kg_BW *S. jambos or T. ananassae*, aqueous extracts. Columns represent means ± SEM. Differences are significant when *P < 0.05

There were differences in blood sugar level modulation in *ob/ob* mice treated with different plant extracts. At 5 wks, the *T. ananassae* (*P* < 0.006) and *S. jambos* (*P* < 0.049) treated mice were significantly more efficient in modulating the effects of the glucose compared to control or *C. speciosus* treated mice (Fig. 4a). All of the plant treated mice had lower baseline blood glucose levels (*S. jambos* BGL 297 mg/dL, *T. ananassae* BGL 262 mg/dL, *C. speciosus* BGL 337 mg/dL) compared to vehicle (BGL 402 mg/dL) treated mice. Moreover, at 10 wks, *S. jambos* (*P* < 0.056) treated mice were more efficient in modulating blood glucose levels when compared with control or mice treated with the remaining plant extracts (Fig. 4b). The baseline blood glucose of *S. jambos* mice was lower (BGL 208 mg/dL) overall. Upon injection of glucose, *S. jambos* treated mice displayed overall lower peaks in blood glucose. There were no differences in the levels of plasma insulin in response to a glucose injection in control, *T. ananassae* or *S. jambos* treated mice (Fig. 5c). In Study 2, the IP-ITT test blood glucose baseline values were lower than in Study 1 and ranged from BGL 221 to 292 mg/dL. By 60 min post-injection glucose concentrations were similar in *S. jambos* (BGL 99 mg/dL) and control (BGL 91 mg/dL) groups. In addition, at 120 min BGL remained at non-diabetic levels (<200 mg/dL). These values were BGL 78 mg/dL (control), BGL 88 mg/dL (*C. speciosus*), BGL 107 mg/dL (*T. ananassae*) and BGL 92 mg/dL (*S. jambos*). Importantly, plasma insulin levels were significantly higher (*P* < 0.02) after the insulin injection in mice regardless of treatment (Fig. 5d).

## Discussion

Globally, as of 2013, an estimated 382 million people have diabetes. In 2012 and 2013 diabetes resulted in 1.5 to 5.1 million deaths per year, making it the 8th leading cause of death worldwide [33]. Diabetes was the third cause (10 %) of deaths in 2010 in Puerto Rico, highlighting the importance of diabetes as a health problem in the Island [34]. Interestingly, traditional medicine users in Puerto Rico rely on the use of herbs to lower blood glucose levels [5]. Thus, in an effort to validate the traditional use of these herbs associated with diabetes management and treatment, we undertook an ethnopharmacological approach to screen commonly used but less studied herbal remedies, their biological activities and possible bioactive compounds. Plant extracts were qualitatively screened by thin layer chromatography (TLC), while flavonoids, phenolics, saponins, tannins and alkaloids were quantified. We also assessed biological activities *in vivo* using two genetic diabetes models.

Our results show that *S. jambos* aqueous extract had significantly higher concentrations of phenolic compounds relative to *T. ananassae* and *C. speciosus*. Phenolics can be classified into phenolic acids (hydroxycinammic acids, phenylpropanoids), flavonoid polyphenolics (flavonoids), and non-flavonoid polyphenolics (tannins). Plant polyphenols are powerful antioxidants that exert their effects either by scavenging reactive oxygen and nitrogen species, or by functioning as chain-breaking peroxyl radical scavengers [35, 36]. Growing evidence shows that excess generation of reactive free radicals, largely due to hyperglycemia, causes oxidative stress in pancreatic β-cells, which further exacerbates the development and progression of diabetes and its complications [37]. Reynertson and collaborators analysis of tropical edible fruits by HPLC-PDA showed that ellagic acid and derivatives were the most abundant phenolic compounds in the methanol-formic acid lyophilized extracts of *S. jambos* fruits [13]. This phenolic compound along with t-cinnamic acid, a phenolic acid found in cinnamon bark, accounted for most of the Total Phenolic Content and antiradical activity of the extracts [38]. Also, several ellagitannins were detected in plant extracts of *S. jambos* from Japan and flavonoids have been isolated from aqueous, methanolic and ethanolic extracts of *S. jambos* leaves among which myricetin and quercetin 3-O-β-D-xylopyranosyl(1–2)-α-L-rhamnopiranoside showed potent anti-inflammatory activity [12]. Although we did not find other published studies that reference the *in vivo* hypoglycemic activity of aqueous extracts of *S. jambos* leaves, an infusion of the leaves of *S. jambos*/*S. cumini* was tested for its anti-diabetic activity by a glucose tolerance test in a randomized, parallel, double-blind clinical trial in non-diabetic and diabetic subjects, with no significant effect in blood glucose levels [39, 40]. *S. cumini* plus placebo tablets, placebo tea plus glyburide tablets (5 mg twice a day), or placebo tea plus placebo tablets were evaluated in these studies. The authors used two different botanical species indistinctively to prepare the teas, thus the activities reported for each botanical species is uncertain. Moreover, *S. cumini* has been thoroughly studied at doses similar to the ones reported in this study for its hypoglycemic, antioxidant and antihyperlipidaemic activities, among others [16, 41]. Again, the pharmacological activities of *S. cumini* have been attributed mainly to the presence of flavonoids and phenolics.

Our studies show that *T. ananassae* had higher concentrations of flavonoids and tannins compared to *S. jambos* and *C. speciosus* extracts. Among the phenolic compounds, the flavonoids, besides functioning as antioxidants, also act as insulin secretagogues that may improve glucose uptake in peripheral tissues. In addition, flavonoids may regulate the activity or expression of rate-limiting enzymes involved in carbohydrate metabolism pathways [42]. Tannins are also phenolic compounds of higher molecular weight commonly used as healing agents. In Ayurveda formulations tannin-rich plants are used for the treatment of diarrhea, while other studies showed that tannins enhance glucose uptake and inhibit adipogenesis [24]. Finally, *C. speciosus* extracts showed a greater amount of alkaloids when compared to *T. ananassae* and *S. jambos*. This could explain the toxicity of *C. speciosus* teas reported in ethnobotanical accounts [4]. Alkaloid fractions have shown beneficial effects in treating hypercholesterolemic, hypertriglyceridemic, hyperlipidemic and/or dyslipidemic conditions and their related complications linked to metabolic disorders such as obesity and diabetes [43]. Phytochemicals reported from *C. speciosus* and other species of the genus *Costus* that have shown hypoglycemic activity and increased plasma insulin concentrations in alloxan-induced diabetic rats include Quercetine glycosides and the pentacyclic triterpene β-Amyrin [44, 45]. An extract of *C. speciosus* was reported to reduce blood glucose concentrations of streptozotocin-induced hyperglycemic rats. A methanolic extract of dried Costus afer Ker Gawl also reduced blood glucose concentrations in streptozotocin induced hyperglycemic rats, stimulating glucose transport in adipocyte cells, suggesting an ability to improve glucose uptake *in vivo* [29].

In the current study, we present the differential effects of *S. jambos*, *T. ananassae* or *C. speciosus* aqueous plant decoctions on glucose levels of two different genetic mouse models of type-2 diabetes. Our first mouse study assessed the efficacy of the plant extracts using a mouse model [C57BLKS/J (*db/db*)] that develops hyperglycemia as young as 6 wks of age [30]. Concentrations of the plant extracts in teas administered to mice were evaluated at a dose smaller (0.2 mg/kg_BW) and commonly consumed by humans (2.2 mg/kg_BW), and at 10 (22 mg/kg_BW) and 100 (220 mg/kg_BW) times greater the concentration used by humans. Our results show that the plant extracts do not affect the weight, food or water intake of *db/db* mice. The glucose peak in non-diabetic individuals rises within one hour of food consumption, but after two hours, blood glucose returns to baseline levels. However, in people with diabetes the glucose peak is higher and it takes longer to decline back to baseline levels upon food consumption. Importantly, studies describe that the experimental basal BGL limit for diabetic animals is 200 mg/dL [8, 46]. Herein we show a severe hyperglycemic animal model where BGL drop to values lower than 200 mg/dL after administration of plant decoctions. Insulin tolerance tests show that mice gavaged with all of the doses of *S. jambos* (0.2, 2.2 and 22 mg/kg_BW), the higher doses of *C. speciosus* (22 and 220 mg/kg_BW), and one dose of *T. ananassae* (2.2 mg/kg_BW) plant extracts tend to modulate glucose better as compared to controls. This suggests that the effect these plants exert in the *db/db* mouse model can be complementary to conventional therapy (insulin injection), rather than when administered as alternative therapy (extract administration alone). Moreover, our data shows that plasma insulin levels are significantly higher after the insulin injection. Since the increase of plasma insulin levels occurs regardless of treatment, it is possible that the mechanistic reason for obtaining lower blood glucose levels in response to *S. jambos* treatment in this mouse model is not due to increases in insulin secretion but to other mechanisms such as lower gluconeogenesis or better glucose uptake by sensitive tissues such as liver, muscle, and adipose tissue. It is known that a balance between insulin secretion and insulin action maintains normal glucose tolerance [47]. The lack of statistical significance of blood glucose modulation in Study 1 could be attributed to the small sample size and to the mouse model chosen. The C57BLKS/J (*db/db*) model is obese via a mutation in the leptin receptor, develops insulin resistance very quickly and has progressive β-cell depletion [29]. This mouse model is commonly used to detect effects of test products in the preservation of β-cell or insulin resistance.

Since in Study 1 we wanted to test a wide range of doses with various plant extracts, the sample size used per treatment was smaller that the one in Study 2. Previous to conducting mouse Study 2, we carried out a statistical power analysis to assess the sample size needed to detect changes in blood glucose taking into account the variability in blood glucose values obtained in Study 1. In Study 2 we used the C57BL/J (*ob/ob*) Type-2 diabetes mouse model. This model is also genetically obese but the mutation lies in the production of leptin [48, 49]. The C57BL/J (*ob/ob*) mice show tendencies in better modulation of euglycemia due to a robust and persistent compensatory pancreatic β-cell response, matching the insulin resistance with hyperinsulinemia. Studies with *ob/ob* mice show that at 4 and 8 wks of age these mice have not developed hyperglycemia [50]. In Study 2, we show that plant extracts, especially *T. ananassae* and *S. jambos*, lowered glucose peaks and kept these glucose peaks stable over the 10 wks trial. In healthy people, blood glucose levels are stable, with the exception of the increased level after meals. Lowering peaks and maintaining stable glucose levels demonstrate the capacity of the body to break glucose, secrete insulin and uptake glucose efficiently. Studies also show that by 15 wks the *ob/ob* mice develop hyperglycemia [30, 50]. In contrast to Study 1, in Study 2 mice treated with the plant extracts or controls have stable non-diabetic glucose values (200 mg/dL or less) at 60 min following an i.p. insulin injection. Also, *ob/ob* mice show plasma insulin levels that are 20 times greater than those detected in *db/db* mice. These results suggest that in the *ob/ob* mice model we are detecting both endogenous and injected insulin levels at the time the IP-ITT was performed. The *ob/ob* mice show mild hyperglycemia that is apparent from 8 to 12  wks of age. At this point, pancreatic β-cell compensation occurs and increased insulin levels are secreted for glucose homeostasis [51]. Our results suggest that the insulin injected is responsible for lowering blood glucose levels in the *ob/ob* mice and not the plant extract. These results differ from that observed in the insulin resistant model. Potential explanations as to why the plant decoctions resulted in mild modulation of BGL include the severity of the insulin resistance associated with these particular animal models. In addition, contrary to other studies that show anti-diabetic efficacy of plant extracts [8, 39, 40, 46, 52] our study aimed to mimic the effects of using traditional remedies by the local Puerto Rican population instead of detecting the activities of these compounds extracted by organic solvents of different polarities [53].

## Conclusions

Our results are the first to show the qualitative and quantitative chemical profile of three commonly used plants by the Puerto Rican population to lower BGL. This report documents for the first time, the *in vivo* mouse studies and the identification and quantification of phenolic compounds, flavonoids, tannins, alkaloids and saponins in *T. ananassae* plant extracts. Moreover, we document a detailed protocol that was used to detect these compounds. Out of the three plants extracts, *S. jambos* shows better *in vivo* efficacy in lowering BGL, when evaluated in diabetes genetic mouse models (*db/db*). At 5 wks, individual mouse BGL readings show that depending on the timepoint either none (90 min), or only one or two animals have BGL > 500 mg/dL. These data substantiate our results that show that *S. jambos* at levels consumed by humans (2.2 mg/kg) tends to modulate BGL better than the rest of the plants. Future studies will evaluate the efficacy of these extracts as complementary therapy. Studies will investigate blood glucose modulation when the extracts are administered in addition to other glucose modulators. Our results demonstrate that in an insulin resistant model (*db/db*) when the extracts are used with insulin (complementary therapy), the plant extracts tend to modulate glucose better than controls, while in *ob/ob* mice this effect is not seen. In some cases, we see that lower doses in complement with insulin have better glucose modulation than the same plant at higher doses (*C. speciosus 22 vs. C. speciosus 220).* It is possible that the plant extracts display a synergistic or additive effect with insulin that is lost by increasing its concentrations. However, without performing proper combinatorial index analysis [54–56]*,* we cannot be certain that such effects indeed occur*.* Furthermore, the study reveals that herbal remedies used as diabetes adjuvants contain anti-diabetic drug principles that require further characterization and studies to assess mode of action to provide a rich source for new drug discovery.

## Additional files

Additional file 1:
**Average food, water intake, and weekly weights in the C57BLKS/J (** ***db/db*** **) mouse model.** Mice orally gavaged with 0.2 mg/kg_BW (a), 2.2 mg/kg_BW (b), 22 mg/kg_BW (c) or 220 mg/kg_BW (d) of *S. jambos, T. ananassae, C. speciosus* or Control for 10 wks were weighed weekly. Mice feed and water were measured daily over 10 wks. Values are mean +/- SEM, n = 4-5/group.ᅟ Additional file 2:
**Average food, water intake, and weekly weights in the C57BL/J (** ***ob/ob*** **) mouse model.** Mice orally gavaged with 2.2 mg/kg_BW of *S. jambos, T. ananassae, C. speciosus* or Control for 10 wks were weighed weekly (a). Mice feed (b) and water (c) were measured daily over 10wks. Values are mean +/- SEM, n = 11-12/group.

## Abbreviations

BGL: Blood glucose levels
GTT: Glucose tolerance test
ITT: Insulin tolerance test
TRAMIL: Traditional medicine in the Islands
wks: Weeks

## References

[1] Geiss LS LY, Kirtland K, Barker L, Burrows NR, Gregg EW (2012). National Diabetes Surveillance System.

[2] Centers for Disease Control and Prevention (2012). Increasing Prevalence of Diagnosed Diabetes — United States and Puerto Rico, 1995–2010. MMWR.

[3] Nuñez E: Plantas Medicinales de Puerto Rico. Río Piedras: Editorial de la Universidad de Puerto Rico; 1989.

[4] Benedetti M (1996). Sembrando y sanando en Puerto Rico.

[5] Alvarado-Guzman JA, Gavillan-Suarez J, Germosen-Robineau L (2009). TRAMIL ethnopharmacological survey: knowledge distribution of medicinal plant use in the southeast region of Puerto Rico. P R Health Sci J.

[6] Sabitha GS A, Patnaik S (2012). *Costus speciosus*, an antidiabetic plant -review. FS J Pharm Res.

[7] Eliza J, Daisy P, Ignacimuthu S (2010). Antioxidant activity of costunolide and eremanthin isolated from *Costus speciosus* (Koen ex. Retz) Sm. Chem Biol Interact.

[8] Eliza J, Daisy P, Ignacimuthu S, Duraipandiyan V (2009). Antidiabetic and antilipidemic effect of eremanthin from *Costus speciosus* (Koen.)Sm., in STZ-induced diabetic rats. Chem Biol Interact.

[9] Murugan S, Uma Devi P, Parameswari NK, Mani KR (2011). Antimicrobial activity of *Syzygium jambos* against selected human pathogens. Int J Pharma Pharmaceut Sci.

[10] Rezende WP, Borges LL, Alves NM, Ferri PH, Paula JR. Chemical variability in the essential oils from leaves of *Syzygium jambos*. Revista Brasileira de Farmacognosia 2013;23(3). http://dx.doi.org/10.1590/S0102-695X2013005000035.

[11] Oliveira AC, Endringer DC, Amorim LA, das Gracas LBM, Coelho MM (2005). Effect of the extracts and fractions of *Baccharis trimera* and *Syzygium cumini* on glycaemia of diabetic and non-diabetic mice. J Ethnopharmacol.

[12] Djipa CD, Delmee M, Quetin-Leclercq J (2000). Antimicrobial activity of bark extracts of *Syzygium jambos* (L.) alston (Myrtaceae). J Ethnopharmacol.

[13] Reynertson KA, Yang H, Jiang B, Basile MJ, Kennelly EJ (2008). Quantitative analysis of antiradical phenolic constituents from fourteen edible Myrtaceae fruits. Food Chem.

[14] Aslan M, Deliorman Orhan D, Orhan N, Sezik E, Yesilada E (2007). *In vivo* antidiabetic and antioxidant potential of *Helichrysum plicatum ssp. plicatum capitulums* in streptozotocin-induced-diabetic rats. J Ethnopharmacol.

[15] Rauter AP, Martins A, Lopes R, Ferreira J, Serralheiro LM, Araujo ME, Borges C, Justino J, Silva FV, Goulart M (2009). Bioactivity studies and chemical profile of the antidiabetic plant *Genista tenera*. J Ethnopharmacol.

[16] Kumar A, Ilavarasan R, Jayachandran T, Decaraman M, Aravindhan P, Padmanabhan N, Krishnan MRV (2009). Phytochemicals Investigation on a Tropical Plant, *Syzygium cumini* from Kattuppalayam, Erode District, Tamil Nadu, South India. Pak J Nutr.

[17] Hernández-Soto RL-C, M.E, Díaz-Jiménez L, Villanueva-Ramírez S. Extracción y cuantificación indirecta de las saponinas de Agave lechuguilla Torrey. In: e-Gnosis*.* vol. 3; 2005; 1-9.

[18] Su HC, Hung LM, Chen JK (2006). Resveratrol, a red wine antioxidant, possesses an insulin-like effect in streptozotocin-induced diabetic rats. Am J Physiol Endocrinol Metab.

[19] Liu X, Kim JK, Li Y, Li J, Liu F, Chen X (2005). Tannic acid stimulates glucose transport and inhibits adipocyte differentiation in 3 T3-L1 cells. J nutr.

[20] Sivitz WI, Yorek MA (2010). Mitochondrial dysfunction in diabetes: from molecular mechanisms to functional significance and therapeutic opportunities. Antioxidants Redox Signal.

[21] Luciano-Montalvo C, Boulogne I, Gavillan-Suarez J (2013). A screening for antimicrobial activities of Caribbean herbal remedies. BMC Complement Altern Med.

[22] Bladt WRS (2009). Plant Drug Analysis. Plant drug analysis: A Thin Layer Chromatography Atlas.

[23] Majaw S, Moirangthem J. Qualitative and Quantitative Analysis of Clerodendron colebrookianum Walp. Leaves and Zingiber cassumunar Roxb. Rhizomes. Ethnobotanical Leaflets. 2009;2009(5):3. Available at: http://opensiuc.lib.siu.edu/ebl/vol2009/iss5/3

[24] Hamuel DJ, Rao DV (2012). Phytochemicals: Extraction Methods, Basic Structures and Mode of Action as Potential Chemotherapeutic Agents. Phytochemicals - A Global Perspective of Their Role in Nutrition and Health.

[25] Pourmorad F, Hosseinimehr SJ, Shahabimajd N (2006). Antioxidant activity, phenol and flavonoid contents of some selected Iranian medicinal plants. Afr J Biotechnol.

[26] Shamsa F, Hamidreza M, Rouhollah G, Mohammadreza V (2008). Spectrophotometric determination of total alkaloids in some Iranian medicinal plants. Thai J Pharm Sci.

[27] AKEaY SA (2010). Phytochemical screening of three medicinal plants: Neem leaf (*Azadirachta indica*), Hibiscus leaf (*Hibiscus rosa-sinensis*) and Spear grass leaf (*Imperata cylindrical*). Continental J Pharmaceut Sci.

[28] Ncube B, Ngunge VN, Finnie JF, Van Staden J (2011). A comparative study of the antimicrobial and phytochemical properties between outdoor grown and micropropagated *Tulbaghia violacea* Harv. plants. J Ethnopharmacol.

[29] Keller AC, Vandebroek I, Liu Y, Balick MJ, Kronenberg F, Kennelly EJ, Brillantes AM (2009). *Costus spicatus* tea failed to improve diabetic progression in C57BLKS/J db/db mice, a model of type 2 diabetes mellitus. J Ethnopharmacol.

[30] Buchanan J, Mazumder PK, Hu P, Chakrabarti G, Roberts MW, Yun UJ, Cooksey RC, Litwin SE, Abel ED (2005). Reduced cardiac efficiency and altered substrate metabolism precedes the onset of hyperglycemia and contractile dysfunction in two mouse models of insulin resistance and obesity. Endocrinology.

[31] Germosén-Robineau LDM, García-González M, Herrera J, Morón F, Sáenz-Campos D, Solís P (2005). Farmacopea Vegetal Caribeña.

[32] National Institutes of Health National Center for Complementary and Integrative Health Policy (2015). NCCIH Policy: Natural Product Integrity.

[33] International Diabetes Federation (2013). IDF Diabetes Atlas.

[34] Ekoé J-M, Zimmet P, Williams R (2001). The Epidemiology of Diabetes Mellitus: An International Perspective.

[35] Evgeny T. Denisov IBAe. Oxidation and Antioxidants in Organic Chemistry and Biology. Boca Raton, Florida, USA, CRC Press; 2005.

[36] Korkina LG, Afanas’ev IB (1997). Antioxidant and chelating properties of flavonoids. Adv Pharmacol.

[37] Johansen JS, Harris AK, Rychly DJ, Ergul A (2005). Oxidative stress and the use of antioxidants in diabetes: linking basic science to clinical practice. Cardiovasc Diabetol.

[38] Jakhetia V, Patel R, Khatri P, Pahuja N, Garg S, Pandey A, Sharma S (2010). Cinnamon: a pharmacological review. J Adv Sci Res.

[39] Teixeira CC, Fuchs FD, Blotta RM, Knijnik J, Delgado IC, Netto MS, Ferreira E, Costa AP, Mussnich DG, Ranquetat GG (1990). Effect of tea prepared from leaves of *Syzygium jambos* on glucose tolerance in nondiabetic subjects. Diabetes Care.

[40] Teixeira CC, Weinert LS, Barbosa DC, Ricken C, Esteves JF, Fuchs FD (2004). *Syzygium cumini* (L.) Skeels in the treatment of type 2 diabetes: results of a randomized, double-blind, double-dummy, controlled trial. Diabetes Care.

[41] Srivastava S, Chandra D (2013). Pharmacological potentials of *Syzygium cumini*: a review. J Sci Food Agric.

[42] Brahmachari G, Tiwari VK MB (2011). Bio-flavonoids with promising anti-diabetic potentials: A critical survey. Opportunity, Challenge and Scope of Natural Products in Medicinal Chemistry.

[43] Hung HY, Qian K, Morris-Natschke SL, Hsu CS, Lee KH (2012). Recent discovery of plant-derived anti-diabetic natural products. Nat Prod Rep.

[44] Jothivel NPS, Appachi M, Singaravel S, Rasilingam D, Deivasigamani K, Thangavel S (2007). Anti-diabetic activity of methanol leaf extract of *Costus pictus* D. Don in alloxan-induced diabetic rats. J Heal Sci.

[45] Mosihuzzaman M, Nahar N, Ali L, Rokeya B, Khan AK, Nur EAM, Nandi RP (1994). Hypoglycemic effects of three plants from eastern Himalayan belt. Diabetes Res.

[46] Rajesh MSHMS, Sathyaprakash RJ, Raghuram Shetty A, Shivananda TN (2009). Antihyperglycemic activity of the various extracts of Costus speciosus rhizomes. J Nat Remedies.

[47] Pratley RE WC, Bogardus C, LeRoith DTS, Olefsky JM (1999). Metabolic abnormalities in the development of type 2 diabetes mellitus. Diabetes Mellitus: A Fundamental and Clinical Text.

[48] Coleman DL (1973). Effects of parabiosis of obese with diabetes and normal mice. Diabetologia.

[49] Coleman DL (2010). A historical perspective on leptin. Nat Med.

[50] Mazumder PK, O’Neill BT, Roberts MW, Buchanan J, Yun UJ, Cooksey RC, Boudina S, Abel ED (2004). Impaired cardiac efficiency and increased fatty acid oxidation in insulin-resistant ob/ob mouse hearts. Diabetes.

[51] Genuth SM, Przybylski RJ, Rosenberg DM (1971). Insulin resistance in genetically obese, hyperglycemic mice. Endocrinology.

[52] Stanely Mainzen Prince P, Kamalakkannan N, Menon VP (2003). *Syzigium cumini* seed extracts reduce tissue damage in diabetic rat brain. J Ethnopharmacol.

[53] World Health Organization. General Guidelines for Methodologies on Research and Evaluation of Traditional Medicine. In*.* Geneva; 2000: 1–73.

[54] Chou TC (2010). Drug combination studies and their synergy quantification using the Chou-Talalay method. Cancer Res.

[55] Prabhakar PK, Doble M (2011). Effect of Natural Products on Commercial Oral Antidiabetic Drugs in Enhancing 2-Deoxyglucose Uptake by 3 T3-L1 Adipocytes. Ther Adv Endocrinol Metab.

[56] Prabhakar PK, Kumar A, Doble M (2014). Combination therapy: a new strategy to manage diabetes and its complications. Phytomedicine.

