# Supplementary figures and images for: Chemical profile and in vivo hypoglycemic effects of Syzygium jambos, Costus speciosus and Tapeinochilos ananassae plant extracts used as diabetes adjuvants in Puerto Rico
Source: BMC Complement Altern Med. 2015 Jul 22;15:244. doi: 10.1186/s12906-015-0772-7 (PMC4511456; doi:10.1186/s12906-015-0772-7)

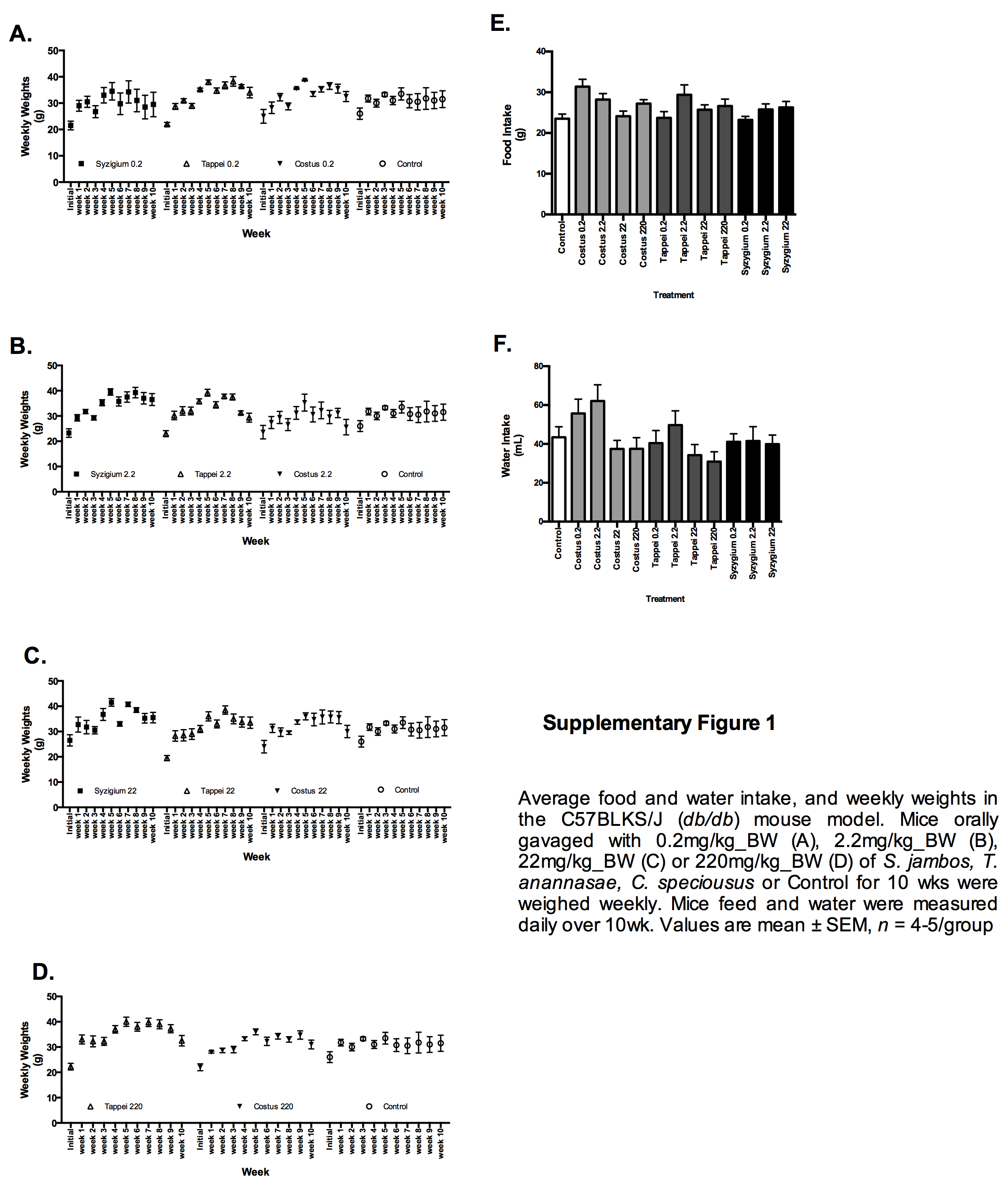

Supplement: Additional file 1: — Average food, water intake, and weekly weights in the C57BLKS/J ( db/db ) mouse model. Mice orally gavaged with 0.2 mg/kg_BW (a), 2.2 mg/kg_BW (b), 22 mg/kg_BW (c) or 220 mg/kg_BW (d) of S. jambos, T. ananassae, C. speciosus or Control for 10 wks were weighed weekly. Mice feed and water were measured daily over 10 wks. Values are mean +/- SEM, n = 4-5/group.ᅟ [file 12906_2015_772_MOESM1_ESM.tiff]

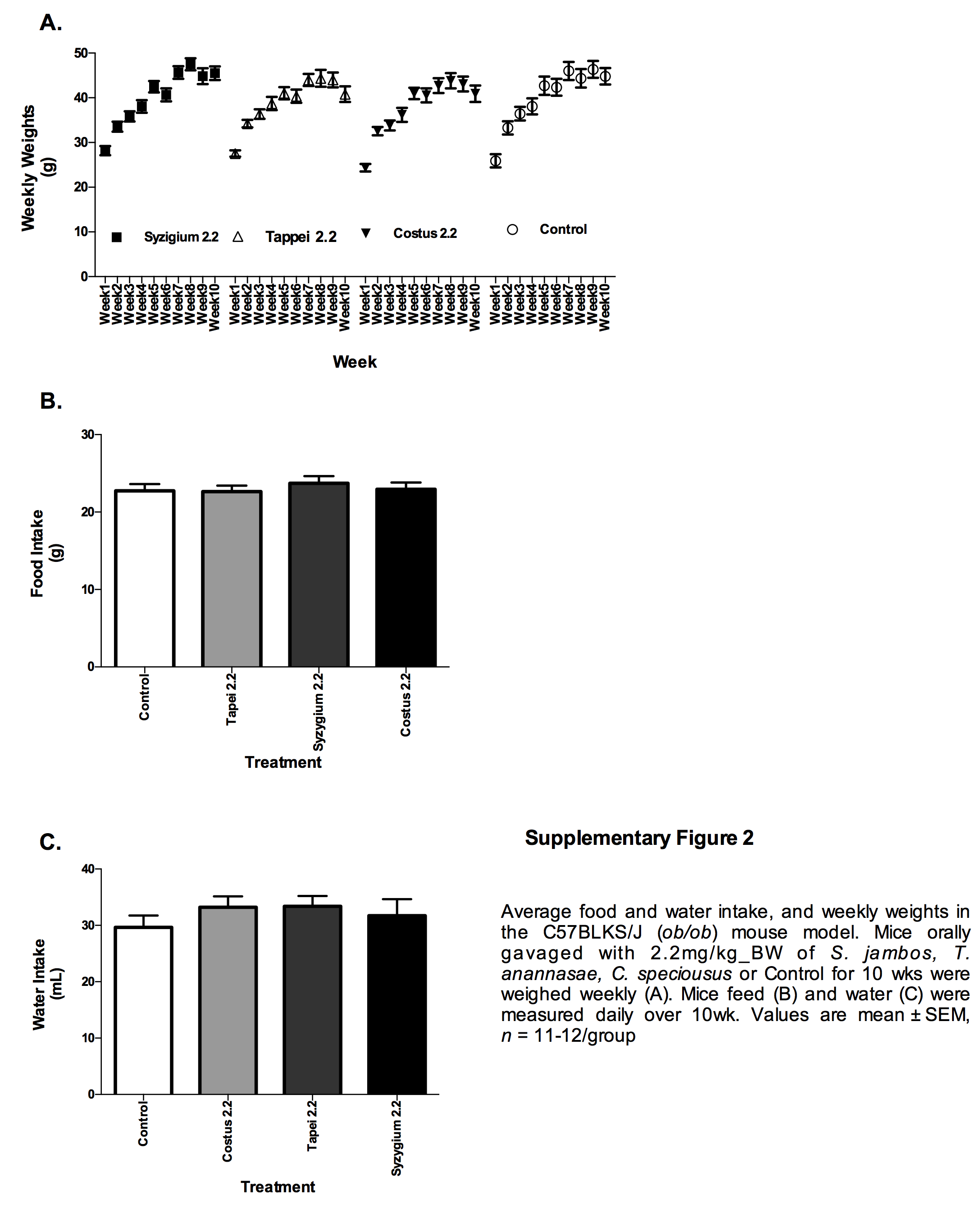

Supplement: Additional file 2: — Average food, water intake, and weekly weights in the C57BL/J ( ob/ob ) mouse model. Mice orally gavaged with 2.2 mg/kg_BW of S. jambos, T. ananassae, C. speciosus or Control for 10 wks were weighed weekly (a). Mice feed (b) and water (c) were measured daily over 10wks. Values are mean +/- SEM, n = 11-12/group. [file 12906_2015_772_MOESM2_ESM.tiff]
